# A Class of Association Measures for Categorical Variables Based on Weighted Minkowski Distance

**DOI:** 10.3390/e21100990

**Published:** 2019-10-11

**Authors:** Qingyang Zhang

**Affiliations:** Department of Mathematical Sciences, University of Arkansas, Fayetteville, AR 72701, USA; qz008@uark.edu

**Keywords:** dependence measure, categorical variable, Minkowski distance, sparse contingency table, total variation distance, mean variance index

## Abstract

Measuring and testing association between categorical variables is one of the long-standing problems in multivariate statistics. In this paper, I define a broad class of association measures for categorical variables based on weighted Minkowski distance. The proposed framework subsumes some important measures including Cramér’s V, distance covariance, total variation distance and a slightly modified mean variance index. In addition, I establish the strong consistency of the defined measures for testing independence in two-way contingency tables, and derive the scaled forms of unweighted measures.

## 1. Introduction

**Figure 1:**
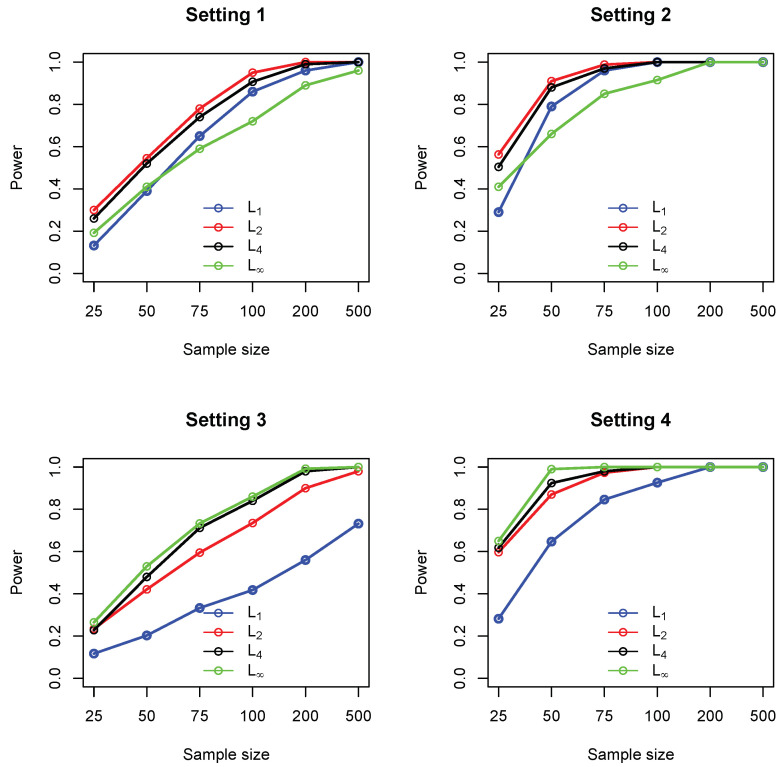
Empirical statistical power of four different measures including $L_{1}$ (blue), $L_{2}$ (red), $L_{4}$ (black) and $L_{\infty }$ (green), under settings 1–4. In each setting, sample sizes are n=25,50,75,100,200,500, and all results were based on 1000 replications.

Measuring and testing the association between categorical variables from observed data is one of the long-standing problems in multivariate statistics. The observed frequencies of two categorical variables are often displayed in a two-way contingency table, and a multinomial distribution can be used to model the cell counts. To be specific, let *X* and *Y* be two categorical random variables with finite sampling spaces X and Y (|X|<∞,|Y|<∞, where |·| stands for the cardinality of a set), and a simple random sample of size *N* can be summarized in a |X|×|Y| table with count $N_{xy}$ in cell (x,y). Let f(x,y), f(x), and f(y) be the joint and marginal probabilities of *X* and *Y*, i.e., f(x,y)=P(X=x,Y=y),f(x)=P(X=x),f(y)=P(Y=y), then the statistical independence between *X* and *Y* can be defined as f(x,y)=f(x)f(y) for any (x,y)∈X×Y, i.e., all joint probabilities equal the product of their marginal probabilities. Pearson’s chi-squared statistic,

$$X^{2}=\sum_{x\in X}\sum_{y\in Y}\frac{{\left(f_{N}\left(x,y\right)-f_{N}\left(x\right)f_{N}\left(y\right)\right)}^{2}}{f_{N}\left(x\right)f_{N}\left(y\right)/N},$$

where $f_{N}\left(x,y\right)=N_{xy}/N$, $f_{N}\left(x\right)=\sum_{y\in Y}N_{xy}/N$, and $f_{N}\left(y\right)=\sum_{x\in X}N_{xy}/N$, has been widely used to test independence in two-way contingency tables. Under independence and sufficient sample size, $X^{2}$ approximately follows a chi-squared distribution with df=(|X|−1)(|Y|−1). However, for insufficient sample size (e.g., $\min_{x,y}N_{x+}N_{+y}/N<5$, where $N_{x+}=\sum_{y\in Y}N_{xy},N_{+y}=\sum_{x\in X}N_{xy}$), the chi-squared test tends to be conservative. Zhang (2019) suggested a random permutation test based on the test statistic

$$D^{2}=\sum_{x\in X}\sum_{y\in Y}{\left(f_{N}\left(x,y\right)-f_{N}\left(x\right)f_{N}\left(y\right)\right)}^{2},$$

which is derived from the squared distance covariance, a measure of the dependence between two random vectors of any type (discrete or continuous) [1,2]. The $D^{2}$ statistic is closely related to Pearson’s chi-squared statistic, both measuring the squared distance between f(x,y) and f(x)f(y), (x,y)∈X×Y. In the numerical study of Zhang (2019), the distance covariance test was evaluated in terms of the statistical power and type I error rate under various settings (see Figures 1–3 in [1]). It is found that for relatively large sample sizes, the distance covariance test performs similarly well as Pearson’s chi-squared test. However, for relatively small sample sizes, the distance covariance test is substantially more powerful and it controls the type I error rate at the nominal level. For small sample, Pearson’s chi-squared test exhibits substantial conservativeness, in the sense that the type I error rate is much lower than the nominal level and it fails to reject many false hypotheses. For instance, in a simulation setting with 20 by 20 table and only 50 samples, the statistical power and type I error rate are both close to zero by Pearson’s chi-squared test, indicating an extreme conservativeness.

Although the distance covariance test has better empirical performance than Pearson’s chi-squared test, especially for small sample size, its theoretical properties have not been investigated. In addition, Zhang (2019) only studied two alternative measures, including distance covariance and projection correlation, but there are many other association measures in the literature remaining unexplored. To name a few, Goodman and Kruskal (1954) introduced two association measures for categorical variables, namely the concentration coefficient and the λ coefficient [3]. Cui et al. (2015) developed a generic association measure based on a mean-variance index [4]. Theil (1970) proposed measuring the association between two categorical variables by the uncertainty coefficient [5]. McCane and Albert (2008) introduced the symbolic covariance, which expresses the covariance between categorical variables in terms of symbolic expressions [6]. In addition, Reshef et al. (2011) proposed a pairwise dependence measure called maximal information coefficient (MIC) based on the grid that maximizes the mutual information gained from the data [7].

The purpose of this paper is to extend my previous work [1] to a broad class of association measures using a general weighted Minkowski distance, and numerically evaluate some selected measures from the proposed class. The proposed class unifies many existing measures including ϕ coefficient, Cramér’s V, distance covariance, total variation distance and a slightly modified mean variance index. Furthermore, the strong consistency of the independence tests based on these measures was established, and the scaled forms of unweighted measures were derived. The proposed class provides a rich set of alternatives to the prevailing chi-squared statistic, and it has many potential applications. For instance, it can be applied to the correlation-based modeling, such as correlation-based deep learning [8]. As enlightened by a reviewer, the proposed method may also be applied to the pseudorandom number generator tests, and may improve some existing chi-squared based tests including the poker test and gap test [9].

The remainder of this paper is structured as follows: In Section 2, I introduce the defined class of association measures, and study some important special cases. The scaled forms of unweighted measures are also derived. Section 3 compares the performance of selected measures using simulated data. Section 4 discusses some extensions including the application to ordinal data and conditional independence test for three-way tables.

## 2. Methods

### 2.1. A Class of Association Measures for Categorical Variables

As the strength of association between two categorical variables can be reflected by the distance between f(x,y) and f(x)f(y), here I define a class of measures based on the weighted Minkowski distance

(1) $$L_{r,\omega }\left(X,Y\right)={\left\{\sum_{x\in X}\sum_{y\in Y}{\left|f\left(x,y\right)-f\left(x\right)f\left(y\right)\right|}^{r}\omega^{r}\left(x,y\right)\right\}}^{\frac{1}{r}},$$

where r≥1, ω(x,y)>0, and ω(x,y) only depends on the marginal distributions of *X* and *Y*. For 0<r<1, the defined distance violates the triangle inequality therefore it is not a metric. However, r=∞ is allowed, and I denote by $L_{\infty ,\omega }\left(X,Y\right)$ the maximum norm. It can be proved that $L_{1,\omega }\left(X,Y\right)\geq L_{2,\omega }\left(X,Y\right)\geq \ldots \geq L_{\infty ,\omega }\left(X,Y\right)$ for a given weight ω(x,y). Throughout this paper, I denote by $L_{r}\left(X,Y\right)$ the unweighted measures, i.e., ω(x,y)=1. The defined class is quite broad and I begin with some important special cases.

Firstly, most of the chi-squared-type measures belong to the defined class. For instance, the ϕ coefficient for 2×2 tables, i.e., |X|=|Y|=2,

$$\phi \left(X,Y\right)={\left\{\sum_{x\in X}\sum_{y\in Y}\frac{{\left|f\left(x,y\right)-f\left(x\right)f\left(y\right)\right|}^{2}}{f(x)f(y)}\right\}}^{\frac{1}{2}},$$

is a special case of $L_{2,\omega }\left(X,Y\right)$, where $\omega \left(x,y\right)={\left\{f\left(x\right)f\left(y\right)\right\}}^{-1/2}$. Extensions of ϕ(X,Y) to I×J tables including Cramér’s *V* and Tschuprow’s *T* [10,11],

$$\begin{array}{cc} V(X,Y) & ={\left\{\sum_{x\in X}\sum_{y\in Y}\frac{{\left|f\left(x,y\right)-f\left(x\right)f\left(y\right)\right|}^{2}}{f(x)f(y)}\right\}}^{\frac{1}{2}}{\left\{\frac{1}{\min(|X|-1,|Y|-1)}\right\}}^{\frac{1}{2}},\\ T(X,Y) & ={\left\{\sum_{x\in X}\sum_{y\in Y}\frac{{\left|f\left(x,y\right)-f\left(x\right)f\left(y\right)\right|}^{2}}{f(x)f(y)}\right\}}^{\frac{1}{2}}{\left\{\frac{1}{\sqrt{\left(|X|-1)(|Y|-1\right)}}\right\}}^{\frac{1}{2}}, \end{array}$$

are also special cases of $L_{2,\omega }\left(X,Y\right)$, where $\omega \left(x,y\right)=\left\{f\left(x\right)f\left(y\right)\min(|X\right|-1,|Y|-{1)\}}^{-1/2}$ for Cramér’s *V*, and $\omega \left(x,y\right)={\left\{f\left(x\right)f\left(y\right)\sqrt{\left(|X|-1)(|Y|-1\right)}\right\}}^{-1/2}$ for Tschuprow’s *T*.

Distance covariance for categorical variables also belongs to the defined class. Distance covariance is a measure of statistical dependence between two random vectors *X* and *Y*. It is a special case of Hilbert-Schmidt independence criterion (HSIC) [12]. Let $\left(X_{1},Y_{1}\right)$, $\left(X_{2},Y_{2}\right)$ and $\left(X_{3},Y_{3}\right)$ be three independent copies of (X,Y), the distance covariance between *X* and *Y* is defined as the square root of

(2) $${\mathrm{dCov}}^{2}\left(X,Y\right)=\mathrm{cov}(∥X_{1}-X_{2}∥,∥Y_{1}-Y_{2}∥)-2\mathrm{cov}(∥X_{1}-X_{2}∥,∥Y_{1}-Y_{3}∥),$$

where ∥·∥ represents distance between vectors, e.g., Euclidean distance. An alternative definition of distance covariance is given in Sejdinovic et al. (2013) [12], which only uses two independent copies of (X,Y). A proof of the equivalency between the two definitions is provided in Appendix A.1. One property of distance covariance is that ${\mathrm{dCov}}^{2}\left(X,Y\right)=0$ if and only if *X* and *Y* are statistically independent, indicating its potential of measuring nonlinear dependence. Zhang (2019) studied the distance covariance for categorical variables under multinomial model. Define $∥X_{1}-X_{2}∥=0$ if $X_{1}=X_{2}$ and 1 otherwise, one can show that

(3) $$\mathrm{dCov}\left(X,Y\right)={\left\{\sum_{x\in X}\sum_{y\in Y}{\left|f\left(x,y\right)-f\left(x\right)f\left(y\right)\right|}^{2}\right\}}^{\frac{1}{2}},$$

and it is easy to see that $\mathrm{dCov}\left(X,Y\right)=L_{2}\left(X,Y\right)$. A detailed proof of Equation (3) is provided in Appendix A.2.

Another special case is total variation distance, which is defined as the largest difference between two probability measures [13]. Let $\mu_{0}\left(\cdot \right)$ and $\mu_{\alpha }\left(\cdot \right)$ be the measures under independence and dependence respectively, the total variation distance between $\mu_{0}$ and $\mu_{\alpha }$ can be used to measure the dependence between variables *X* and *Y*

(4) $$\delta \left(\mu_{0},\mu_{\alpha }\right)=\max_{S\subset X\times Y}\left|\mu_{0}\left(S\right)-\mu_{\alpha }\left(S\right)\right|.$$

In the case of discrete sampling spaces, let $S^{+}=\left\{\left(x,y\right),s.t.,f\left(x,y\right)>f\left(x\right)f\left(y\right)\right\}$ and $S^{-}=\left\{\left(x,y\right),s.t.,f\left(x,y\right)<f\left(x\right)f\left(y\right)\right\}$, then we have

(5) $$\delta \left(\mu_{0},\mu_{\alpha }\right)=\;|\mu_{0}\left(S^{+}\right)-\mu_{\alpha }\left(S^{+}\right)\left|\;=\;\right|\mu_{0}\left(S^{-}\right)-\mu_{\alpha }\left(S^{-}\right)|\;=\frac{1}{2}\sum_{x\in X}\sum_{y\in Y}\left|f\left(x,y\right)-f\left(x\right)f\left(y\right)\right|,$$

therefore $\delta \left(\mu_{0},\mu_{\alpha }\right)=L_{1,\omega }\left(X,Y\right)$, where $\omega \left(X,Y\right)=\frac{1}{2}$.

In addition, I pointed out that the mean variance index (MV) recently developed by Cui et al. [4] also belongs to our defined class, subject to some slight modifications. The MV between two variables *X* and *Y* is defined as $MV\left(X|Y\right)=E_{X}\left(V_{Y}\left(F\left(X|Y\right)\right)\right)$, where F(x|y) stands for conditional distribution function. It can be proved that MV(X|Y)=0 if and only if *X* and *Y* are independent. The MV measure is originally developed for continuous variables. To make it suitable for categorical variables while maintaining the main theoretical property, I slightly modified the definition of MV. First, I replaced the conditional c.d.f. F(x|y) with conditional p.m.f. f(x|y). Second, as the MV measure is generally asymmetric, i.e., MV(X|Y)≠MV(Y|X), I considered a symmetric version of the index, $MV\left(X,Y\right)=\frac{1}{2}\left(MV\left(X|Y\right)+MV\left(Y|X\right)\right)$. With the two modifications, one can prove the following result (a detailed proof is provided in Appendix A.3)

$$\sqrt{\mathrm{MV}(X,Y)}={\left\{\sum_{x\in X}\sum_{y\in Y}\frac{1}{2}{\left|f\left(x,y\right)-f\left(x\right)f\left(y\right)\right|}^{2}\left(\frac{f(x)}{f(y)}+\frac{f(y)}{f(x)}\right)\right\}}^{\frac{1}{2}},$$

therefore $\sqrt{\mathrm{MV}(X,Y)}=L_{2,\omega }\left(X,Y\right)$, where $\omega \left(x,y\right)=\sqrt{\frac{1}{2}\left(\frac{f(x)}{f(y)}+\frac{f(y)}{f(x)}\right)}$. As $\frac{1}{2}\left(\frac{f(x)}{f(y)}+\frac{f(y)}{f(x)}\right)\geq 1$, we also have $\sqrt{\mathrm{MV}(X,Y)}\geq L_{2}\left(X,Y\right)$.

Similar as the MV index, the symmetric version of some other directional association measures (e.g., the concentration coefficient [3]), are also the special cases of $L_{r,\omega }$.

### 2.2. Sample Estimate and Independence Test

Given a simple random sample of size *N*, one can estimate $L_{r,\omega ,N}\left(X,Y\right)$ using sample quantities

(6) $$L_{r,\omega ,N}\left(X,Y\right)={\left\{\sum_{x\in X}\sum_{y\in Y}{\left|f_{N}\left(x,y\right)-f_{N}\left(x\right)f_{N}\left(y\right)\right|}^{r}\omega_{N}^{r}\left(x,y\right)\right\}}^{\frac{1}{r}},$$

where $f_{N}\left(x,y\right)$, $f_{N}\left(x\right)$ and $f_{N}\left(y\right)$ represent the maximum likelihood estimates of joint and marginal probabilities, respectively, i.e., $f_{N}\left(x,y\right)=N_{xy}/N$, $f_{N}\left(x\right)=\sum_{y\in Y}N_{xy}/N$, and $f_{N}\left(y\right)=\sum_{x\in X}N_{xy}/N$. The following theorem establishes the strong consistency of the independence test based on $L_{r,\omega ,N}\left(X,Y\right)$ (a detailed proof is provided in Appendix A.4):

**Theorem** **1.** *Assume that the estimated weights are bounded above by a constant C>0, i.e., $\sup_{x,y}\omega_{N}\left(x,y\right)=C$, then for any r≥1 and ϵ>0, we have $P\left(L_{r,\omega ,N}\left(X,Y\right)>\epsilon \right)<\left(2^{\left|X||Y\right|}+2^{\left|Y\right|}+2^{\left|X\right|}\right)\exp\left(-N\epsilon^{2}/18C^{2}\right)$ under independence. The inequality also holds for maximum norm $L_{\infty ,\omega ,N}\left(X,Y\right)$.*

It is noteworthy that the asymptotic null distribution of $L_{r,\omega ,N}\left(X,Y\right)$ is impratical to derive. The theorem above provides a simple way to compute the upper bound of *p*-value, however, the bound $\left(2^{\left|X||Y\right|}+2^{\left|Y\right|}+2^{\left|X\right|}\right)\exp\left(-N\epsilon^{2}/18C^{2}\right)$ is generally not tight, thus the *p*-value could be largely overestimated. Here, I suggest a simple permutation procedure to evaluate the significance. One can randomly shuffle the observations of *X* (or equivalently, the observations of *Y*) for *M* times, and compute the test statistic $L_{r,\omega ,N}\left(X_{perm},Y\right)$ for each permuted dataset. The permutation *p*-value can be computed as the proportion of $L_{r,\omega ,N}\left(X_{perm},Y\right)$’s that exceed the actually observed one. I used the permutation *p*-value to evaluate statistical significant in our simulation studies.

### 2.3. Scaled Forms of Unweighted Measures

Motivated by the classic correlation coefficient, I define the following scaled form for unweighted measure $L_{r}\left(X,Y\right)$:

(7) $$L_{r}^{*}\left(X,Y\right)=\frac{L_{r}\left(X,Y\right)}{\sqrt{L_{r}\left(X,X\right)}\sqrt{L_{r}\left(Y,Y\right)}},$$

where $L_{r}\left(X,X\right)={\left\{\sum_{x\in X}\sum_{x^{\prime }\in X}{\left|f\left(x,x^{\prime }\right)-f\left(x\right)f\left(x^{\prime }\right)\right|}^{r}\right\}}^{\frac{1}{r}}$, $f\left(x,x^{\prime }\right)=f\left(x\right)$ if $x=x^{\prime }$ and $f(x,x^{\prime })=0$ otherwise.

The term $L_{r}\left(X,X\right)$ can be written as

$$L_{r}\left(X,X\right)={\left\{\sum_{x\in X}|f\left(x\right)-f^{2}{\left(x\right)|}^{r}+\sum_{x\in X}\sum_{x^{\prime }\in X\setminus x}{\left|f\left(x\right)f\left(x^{\prime }\right)\right|}^{r}\right\}}^{\frac{1}{r}},$$

and as examples, the explicit expressions for $L_{1}\left(X,X\right)$, $L_{2}\left(X,X\right)$, and $L_{\infty }\left(X,X\right)$ are given below

- $L_{1}\left(X,X\right)=\sum_{x\in X}\left[f\left(x\right)-f^{2}\left(x\right)\right]+\sum_{x\in X}\sum_{x^{\prime }\in X\setminus x}\left[f\left(x\right)f\left(x^{\prime }\right)\right]=2\left[1-\sum_{x\in X}f^{2}\left(x\right)\right]$
- $L_{2}\left(X,X\right)={\left\{\sum_{x\in X}f^{2}\left(x\right)\left[\sum_{x\in X}f^{2}\left(x\right)+1\right]-2\sum_{x\in X}f^{3}\left(x\right)\right\}}^{\frac{1}{2}}$
- $L_{\infty }\left(X,X\right)=\max_{x\in X}\left[f\left(x\right)-f^{2}\left(x\right)\right]\vee \max_{x\in X,x^{\prime }\in X\setminus x}\left[f\left(x\right)f\left(x^{\prime }\right)\right]=\max_{x\in X}\left[f\left(x\right)-f^{2}\left(x\right)\right]$

It can be seen that $L_{2}^{*}\left(X,Y\right)$ is same as the distance correlation between *X* and *Y* [1], therefore $0\leq L_{2}^{*}\left(X,Y\right)\leq 1$, where $L_{r}^{*}\left(X,Y\right)=0$ if and only if *X* and *Y* are independent. In fact, for any 1≤r<∞, if f(x)>0,f(y)>0 for x∈X,y∈Y, it can be proved that $0\leq L_{r}^{*}\left(X,Y\right)\leq 1$, where $L_{r}^{*}\left(X,Y\right)=0$ if and only if *X* and *Y* are independent, and $L_{r}^{*}\left(X,Y\right)=1$ if and only if *X* and *Y* have perfect association, i.e., |X|=|Y| and for any x∈X, there exists a unique y∈Y, such that f(x,y)=f(x)=f(y).

For $L_{\infty }^{*}\left(X,Y\right)$, by Cauchy-Schwarz inequality,

$$\begin{array}{cc} L_{\infty }\left(X,Y\right) & =\max_{x\in X,y\in Y}\left|f\left(x,y\right)-f\left(x\right)f\left(y\right)\right|\\ \; & =\max_{x\in X,y\in Y}\left|cov\left(I\left\{X=x\right\},I\left\{Y=y\right\}\right)\right|\\ \; & \leq \max_{x\in X,y\in Y}\sqrt{V(I\{X=x\})V(I\{Y=y\})}\\ \; & =\max_{x\in X}\sqrt{V(I\{X=x\})}\max_{y\in Y}\sqrt{V(I\{Y=y\})}\\ \; & =\sqrt{\max_{x\in X}\left[f\left(x\right)-f^{2}\left(x\right)\right]}\sqrt{\max_{y\in Y}\left[f\left(y\right)-f^{2}\left(y\right)\right]}\\ \; & =\sqrt{L_{\infty }\left(X,X\right)L_{\infty }\left(Y,Y\right)}, \end{array}$$

therefore $0\leq L_{\infty }^{*}\leq 1$. However, in general, $L_{\infty }^{*}\left(X,Y\right)=1$ does not imply that *X* and *Y* are perfectly associated. I gave an example in Table 1, where $L_{\infty }^{*}\left(X,Y\right)=1$ but *X* and *Y* are not perfectly associated.

## 3. Numerical Study

Two simulation studies were conducted to compare the performance of some selected measures from our defined class. In both simulations, I set |X|=|Y|=10 and varied the sample size from 25 to 500, so that the simulated contingency tables were relatively large and sparse (average count N/|X||Y| is between 0.25 and 5).

In the first simulation study, I considered the independence test based on different unweighted measures, including $L_{1}$, $L_{2}$, $L_{4}$ and $L_{\infty }$, under the following multinomial settings:

- Setting 1: f(x,y)=0.05 for 10 randomly selected cells and $f\left(x,y\right)=\frac{0.5}{90}$ for the remaining 90 cells
- Setting 2: f(x,y)=0.08 for 10 randomly selected cells and $f\left(x,y\right)=\frac{0.2}{90}$ for the remaining 90 cells
- Setting 3: f(x,y)=0.1 for one randomly selected cell and $f\left(x,y\right)=\frac{0.9}{99}$ for the remaining 99 cells
- Setting 4: f(x,y)=0.2 for one randomly selected cell and $f\left(x,y\right)=\frac{0.8}{99}$ for the remaining 99 cells

**Figure 2:**
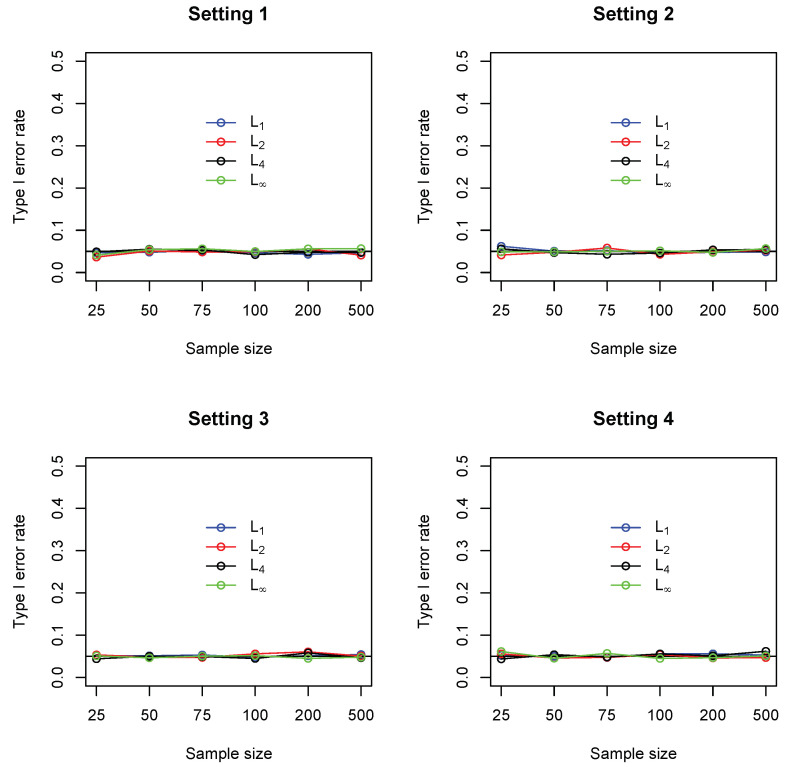
Empirical Type I error rate of four different measures including $L_{1}$ (blue), $L_{2}$ (red), $L_{4}$ (black) and $L_{\infty }$ (green), under settings 1–4. In each setting, sample sizes are n=25,50,75,100,200,500, and all results were based on 1000 replications.

For each test, the *p*-values were computed based on 2000 random permutations. Figure 1 summarizes the empirical statistical power of the four tests under significance level 0.05. It could be seen that, in settings 1 and 2, the $L_{2}$ measure (Euclidean distance) performed consistently better than the other three (comparable to $L_{4}$). The maximum norm $L_{\infty }$ performs the worst in these two settings. In settings 3 and 4, where a single cell accounts for most deviation from independence, the maximum norm performs the best, while the $L_{1}$ measure (Manhattan distance) gives the lowest power. Figure 2 summarizes the type I error rate, where it can be seen that all the four tests control the type I error rates at the nominal level of 0.05.

**Figure 3:**
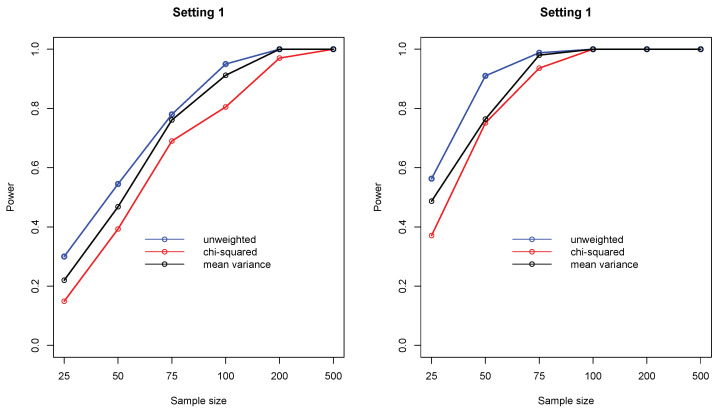
Empirical statistical power of three $L_{2,\omega }$ measures including ω(x,y)=1 (distance covariance), $\omega \left(x,y\right)={\left\{f\left(x\right)f\left(y\right)\right\}}^{-1/2}$ (chi-squared), and $\omega \left(x,y\right)=\sqrt{\frac{1}{2}\left(\frac{f(x)}{f(y)}+\frac{f(y)}{f(x)}\right)}$ (symmetric mean variance index), under settings 1 and 2. In each setting, sample sizes are n=25,50,75,100,200,500, and all results were based on 1000 replications.

In the second simulation study, I focused on $L_{2,\omega }\left(X,Y\right)$ as it subsumes many popular measures. In particular, I compared three different weight functions, including ω(x,y)=1 (distance covariance), $\omega \left(x,y\right)={\left\{f\left(x\right)f\left(y\right)\right\}}^{-1/2}$ (Pearson’s chi-squared), and $\omega \left(x,y\right)=\sqrt{\frac{1}{2}\left(\frac{f(x)}{f(y)}+\frac{f(y)}{f(x)}\right)}$ (modified mean variance index). Figure 3 shows the empirical statistical power of the three measures under settings 1 and 2, where it can be seen that the unweighted $L_{2}$ compares favorably to the weighted ones.

Based on the simulation studies, I recommend to the unweighted $L_{r}$ measures with a moderate choice of *r*, for instance, r=2,3,4 for large sparse tables, because they could give satisfactory and stable statistical power in general scenarios. The maximum norm $L_{\infty }$ is not recommended, unless one is very confident that there exist a very small number of cells that account for most deviation from independence.

## 4. Discussion

In this work, I proposed a rich class of dependence measures for categorical variables based on weighted Minkowski distance. The defined class unifies a number of existing measures including Cramér’s V, distance covariance, total variation distance and a slightly modified mean variance index. I provided the scaled forms of unweighted measures, which range from 0 (independence) to 1 (perfect association). Further, I established the strong consistency of the defined measures and suggested a simple permutation test for evaluating significance. Although I have used nominal and univariate categorical variables for illustrations, the proposed framework can be extended to other data types and problems:

First, the proposed measures can be used to detect ordinal association by assigning proper weights. Similar as Pearson’s correlation coefficient, one may assign larger weights to more extreme categories of *X* and *Y*. To be specific, let $d(x,x^{\prime })$ be the predefined distance between categories X=x and $X=x^{\prime }$, and $d(y,y^{\prime })$ be the distance between *y* and $y^{\prime }$, and one could apply the following weight function

$$\omega \left(x,y\right)=E\left(d\left(x,X\right)d\left(y,Y\right)\right)=\sum_{x^{\prime }\in X\setminus x,y^{\prime }\in Y\setminus y}d\left(x,x^{\prime }\right)d\left(y,y^{\prime }\right)f\left(x^{\prime }\right)f\left(y^{\prime }\right),$$

which assigns larger weights to cells in the corners but smaller weights to cells in the center of the table.

Second, my framework can be generalized to random vectors and multi-way tables. In the case of three-way table (X,Y,Z), one can define the following Minkowski distance between f(x,y,z) and f(x,y)f(z)

$$L_{r,\omega }\left(\left(X,Y\right),Z\right)={\left\{\sum_{x\in X}\sum_{y\in Y}\sum_{z\in Z}{\left|f\left(x,y,z\right)-f\left(x,y\right)f\left(z\right)\right|}^{r}\omega^{r}\left(x,y,z\right)\right\}}^{\frac{1}{r}},$$

which can be used to test the joint independence between (X,Y) and *Z*, or equivalently, to test the homogeneity of the joint distribution of (X,Y) at different levels of *Z*. A similar permutation procedure can be applied to evaluate the statistical significance. One can also define the distance between f(x,y,z) and f(x)f(y)f(z) to test the mutual independence of (X,Y,Z)

$$L_{r,\omega }\left(X,Y,Z\right)={\left\{\sum_{x\in X}\sum_{y\in Y}\sum_{z\in Z}{\left|f\left(x,y,z\right)-f\left(x\right)f\left(y\right)f\left(z\right)\right|}^{r}\omega^{r}\left(x,y,z\right)\right\}}^{\frac{1}{r}},$$

Furthermore, the framework can be extended to conditional independence test in three-way tables [14], by defining distance between conditional joint probabilities f(x,y|z) and the product of conditional marginal probabilities f(x|z)f(y|z)

$$L_{r,\omega }\left(X,Y|Z\right)={\left\{\sum_{x\in X}\sum_{y\in Y}\sum_{z\in Z}{\left|f\left(x,y|z\right)-f\left(x|z\right)f\left(y|z\right)\right|}^{r}\omega^{r}\left(x,y,z\right)\right\}}^{\frac{1}{r}}.$$

## Figures and Tables

**Table 1:** An example that *X* and *Y* are not perfectly associated, but $L_{\infty }^{*}\left(X,Y\right)=1$.

	*Y* = 1	*Y* = 2	*Y* = 3
*X* = 1	1/2	0	0
*X* = 2	0	1/8	1/8
*X* = 3	0	1/8	1/8

## Abbreviations

The following abbreviations are used in this manuscript:

MIC	maximum information coefficient
HSIC	Hilbert-Schmidt independence criterion
MV	mean variance index
c.d.f.	cumulative distribution function
p.m.f.	probability mass function

## Appendix A. Technical Details

### Appendix A.1. Equivalency between Two Definitions of Distance Covariance

- Definition by Szekely et al. (2007): ${\mathrm{dCov}}^{2}\left(X,Y\right)=E_{X_{1}X_{2}Y_{1}Y_{2}}(∥X_{1}-X_{2}∥∥Y_{1}-Y_{2}∥)+E_{X_{1}X_{2}}(∥X_{1}-X_{2}∥)E_{Y_{1}Y_{2}}(∥Y_{1}-Y_{2}∥)-2E_{X_{1}X_{2}Y_{1}Y_{3}}(∥X_{1}-X_{2}∥∥Y_{1}-Y_{3}∥)$
- Definition by Sejdinovic et al. (2013): ${\mathrm{dCov}}^{2}\left(X,Y\right)=E_{X_{1}X_{2}Y_{1}Y_{2}}(∥X_{1}-X_{2}∥∥Y_{1}-Y_{2}∥)+E_{X_{1}X_{2}}(∥X_{1}-X_{2}∥)E_{Y_{1}Y_{2}}(∥Y_{1}-Y_{2}∥)-2E_{X_{1}Y_{1}}(E_{X_{2}}(∥X_{1}-X_{2}∥)E_{Y_{2}}(∥Y_{1}-Y_{2}∥))$

The first two terms are the same, and the equivalency between the two definitions can be showed as follows:

$$\begin{array}{cc} \; & E_{X_{1}Y_{1}}(E_{X_{2}}(∥X_{1}-X_{2}∥)E_{Y_{2}}(∥Y_{1}-Y_{2}∥))\\ \; & =\int_{x_{1}}\int_{y_{1}}\left[\int_{x_{2}}∥x_{1}-x_{2}∥f\left(x_{2}\right)dx_{2}\int_{y_{2}}∥y_{1}-y_{2}∥f\left(y_{2}\right)dy_{2}\right]f\left(x_{1},y_{1}\right)dx_{1}dy_{1}\\ \; & =\int_{x_{1}}\int_{y_{1}}\left[\int_{x_{2}}∥x_{1}-x_{2}∥f\left(x_{2}\right)dx_{2}\int_{y_{3}}∥y_{1}-y_{3}∥f\left(y_{3}\right)dy_{3}\right]f\left(x_{1},y_{1}\right)dx_{1}dy_{1}\\ \; & =\int_{x_{1}}\int_{x_{2}}\int_{y_{1}}\int_{y_{3}}∥x_{1}-x_{2}∥∥y_{1}-y_{3}∥f\left(x_{1},y_{1}\right)f\left(x_{2}\right)f\left(y_{3}\right)dx_{1}dx_{2}dy_{1}dy_{3}\\ \; & =E_{X_{1}X_{2}Y_{1}Y_{3}}(∥X_{1}-X_{2}∥∥Y_{1}-Y_{3}∥) \end{array}$$

### Appendix A.2. Derivation of Equation (3)

Following Zhang (2019), I rewrite categorical variables *X* and *Y* as two random vectors of dimensions |X| and |Y|, $X={\left\{I\left(X=x\right)\right\}}_{x\in X}$ and $Y={\left\{I\left(Y=y\right)\right\}}_{y\in Y}$, where I(·) stands for the indicator function. Let $∥X_{1}-X_{2}∥$ equal 0 if $X_{1}=X_{2}$ and 1 otherwise. Let $\left(X_{1},Y_{2}\right)$, $\left(X_{2},Y_{2}\right)$, $\left(X_{3},Y_{3}\right)$ be three independent copies of (X,Y). By Equation (2), the squared distance covariance can be also expressed as

$${\mathrm{dCov}}^{2}\left(X,Y\right)=E(∥X_{1}-X_{2}∥∥Y_{1}-Y_{2}∥)+E(∥X_{1}-X_{2}∥)E(∥Y_{1}-Y_{2}∥)-2E(∥X_{1}-X_{2}∥∥Y_{1}-Y_{3}∥).$$

Under multinomial sampling scheme, it is straightforward to show that

$$\begin{array}{cc} E(∥X_{1}-X_{2}∥∥Y_{1}-Y_{2}∥) & =P\left(X_{1}\neq X_{2},Y_{1}\neq Y_{2}\right)=\sum_{x\in X}\sum_{y\in Y}f\left(x,y\right)\left[1-f\left(x\right)-f\left(y\right)+f\left(x,y\right)\right],\\ E(∥X_{1}-X_{2}∥)E(∥Y_{1}-Y_{2}∥) & =P\left(X_{1}\neq X_{2}\right)P\left(Y_{1}\neq Y_{2}\right)=\left[1-\sum_{x\in X}f^{2}\left(x\right)\right]\left[1-\sum_{y\in Y}f^{2}\left(y\right)\right],\\ E(∥X_{1}-X_{2}∥∥Y_{1}-Y_{3}∥) & =P\left(X_{1}\neq X_{2},Y_{1}\neq Y_{3}\right)=\sum_{x\in X}\sum_{y\in Y}f\left(x,y\right)\left[1-f\left(x\right)\right]\left[1-f\left(y\right)\right]. \end{array}$$

Summarizing the results above, I have

$$\mathrm{dCov}\left(X,Y\right)={\left\{\sum_{x\in X}\sum_{y\in Y}{\left|f\left(x,y\right)-f\left(x\right)f\left(y\right)\right|}^{2}\right\}}^{\frac{1}{2}}.$$

### Appendix A.3. Derivation of the Modified Mean Variance Index

The symmetric mean variance index is defined as

$$MV\left(X,Y\right)=\frac{1}{2}\left(MV\left(X|Y\right)+MV\left(Y|X\right)\right)=\frac{1}{2}\left(E_{X}\left(V_{Y}\left(f\left(X|Y\right)\right)\right)+E_{Y}\left(V_{X}\left(f\left(Y|X\right)\right)\right)\right).$$

I first derived the explicit formula for $E_{X}\left(V_{Y}\left(f\left(X|Y\right)\right)\right)$:

$$\begin{array}{cc} E_{X}\left(V_{Y}\left(f\left(X|Y\right)\right)\right) & =E_{X}E_{Y}{\left(f\left(X|Y\right)\right)}^{2}-E_{X}E_{Y}^{2}\left(f\left(X|Y\right)\right)\\ \; & =\sum_{x\in X}\sum_{y\in Y}f^{2}\left(x,y\right)\frac{f(x)}{f(y)}-\sum_{x\in X}f^{3}\left(x\right)\\ \; & =\sum_{x\in X}\sum_{y\in Y}\frac{f(x)}{f(y)}\left(f^{2}\left(x,y\right)-f^{2}\left(x\right)f^{2}\left(y\right)\right)\\ \; & =\sum_{x\in X}\sum_{y\in Y}\frac{f(x)}{f(y)}\left\{{\left(f\left(x,y\right)-f\left(x\right)f\left(y\right)\right)}^{2}-2f^{2}\left(x\right)f^{2}\left(y\right)+2f\left(x,y\right)f\left(x\right)f\left(y\right)\right\}\\ \; & =\sum_{x\in X}\sum_{y\in Y}\frac{f(x)}{f(y)}{\left(f\left(x,y\right)-f\left(x\right)f\left(y\right)\right)}^{2} \end{array}$$

Similarly, it can seen that $E_{Y}\left(V_{X}\left(f\left(Y|X\right)\right)\right)=\sum_{x\in X}\sum_{y\in Y}\frac{f(y)}{f(x)}{\left(f\left(x,y\right)-f\left(x\right)f\left(y\right)\right)}^{2}$, therefore

$$MV\left(X,Y\right)=\sum_{x\in X}\sum_{y\in Y}\frac{1}{2}\left(\frac{f(y)}{f(x)}+\frac{f(x)}{f(y)}\right){\left(f\left(x,y\right)-f\left(x\right)f\left(y\right)\right)}^{2}.$$

### Appendix A.4. Proof of Theorem 1

Because $L_{1,\omega ,N}\geq L_{2,\omega ,N}\geq \ldots \geq L_{\infty ,\omega ,N}$, I only need prove the strong consistency for $L_{1,\omega ,N}$. For categorical variable *X*, let $f{\left(x\right)}_{x\in X}$ be the probability mass function, *N* be the sample size, and $f_{N}\left(x\right)$ be the sample estimate, Biau and Gyorfi (2005) [15] proved the following result

**Lemma** **A1.**
*For any ϵ>0, $P(\sum_{x\in X}|f_{N}\left(x\right)-f\left(x\right)|>\epsilon )<2^{\left|X\right|}e^{-\frac{N\epsilon^{2}}{2}}$.*

As $\sup_{x,y}\omega \left(x,y\right)=C>0$, I have

$$L_{1,\omega ,N}\left(X,Y\right)\leq C(\sum_{x\in X}\sum_{y\in Y}|f_{N}\left(x,y\right)-f\left(x,y\right)|+\sum_{x\in X}\sum_{y\in Y}|f\left(x,y\right)-f\left(x\right)f\left(y\right)|+\sum_{x\in X}\sum_{y\in Y}|f_{N}\left(x\right)f_{N}\left(y\right)-f\left(x\right)f\left(y\right)\left|\right).$$

Under independence, I have $\sum_{x\in X}\sum_{y\in Y}\left|f\left(x,y\right)-f\left(x\right)f\left(y\right)\right|=0$. By Lemma A1, the first term $\sum_{x\in X}\sum_{y\in Y}\left|f_{N}\left(x,y\right)-f\left(x,y\right)\right|$ satisfies that

$$P\left(C\sum_{x\in X}\sum_{y\in Y}\left|f_{N}\left(x,y\right)-f\left(x,y\right)\right|>\frac{\epsilon }{3}\right)<2^{\left|X||Y\right|}e^{-\frac{N\epsilon^{2}}{18C^{2}}},$$

The third term can be bounded as follows:

$$\begin{array}{cc} \sum_{x\in X}\sum_{y\in Y}\left|f_{N}\left(x\right)f_{N}\left(y\right)-f\left(x\right)f\left(y\right)\right| & \leq \sum_{x\in X}\sum_{y\in Y}|f\left(x\right)f_{N}\left(y\right)-f\left(x\right)f\left(y\right)|+\sum_{x\in X}\sum_{y\in Y}\left|f_{N}\left(x\right)f_{N}\left(y\right)-f\left(x\right)f_{N}\left(y\right)\right|\\ \; & =\sum_{y\in Y}|f_{N}\left(y\right)-f\left(y\right)|+\sum_{x\in X}\left|f_{N}\left(x\right)-f\left(x\right)\right| \end{array}$$

By Lemma A1, I have

$$P\left(C\sum_{y\in Y}\left|f_{N}\left(y\right)-f\left(y\right)\right|>\frac{\epsilon }{3}\right)<2^{\left|Y\right|}e^{-\frac{N\epsilon^{2}}{18C^{2}}},$$

$$P\left(C\sum_{x\in X}\left|f_{N}\left(x\right)-f\left(x\right)\right|>\frac{\epsilon }{3}\right)<2^{\left|X\right|}e^{-\frac{N\epsilon^{2}}{18C^{2}}},$$

and summarizing the results above, I have

$$\begin{array}{cc} P\left(L_{1,\omega ,N}\left(X,Y\right)>\epsilon \right) & \leq P\left(C\sum_{x\in X}\sum_{y\in Y}\left|f_{N}\left(x,y\right)-f\left(x,y\right)\right|>\frac{\epsilon }{3}\right)\\ \; & +P\left(C\sum_{y\in Y}\left|f_{N}\left(y\right)-f\left(y\right)\right|>\frac{\epsilon }{3}\right)+P\left(C\sum_{x\in X}\left|f_{N}\left(x\right)-f\left(x\right)\right|>\frac{\epsilon }{3}\right)\\ \; & <2^{\left|X||Y\right|}e^{-\frac{N\epsilon^{2}}{18C^{2}}}+2^{\left|Y\right|}e^{-\frac{N\epsilon^{2}}{18C^{2}}}+2^{\left|X\right|}e^{-\frac{N\epsilon^{2}}{18C^{2}}}\\ \; & =\left(2^{\left|X||Y\right|}+2^{\left|X\right|}+2^{\left|Y\right|}\right)e^{-\frac{N\epsilon^{2}}{18C^{2}}}. \end{array}$$
